# X-ray crystallographic studies of RoAb13 bound to PIYDIN, a part of the N-terminal domain of C-C chemokine receptor 5

**DOI:** 10.1107/S2052252521005340

**Published:** 2021-07-01

**Authors:** Lata Govada, Emmanuel Saridakis, Sean C. Kassen, Ahmad Bin-Ramzi, Rhodri Marc Morgan, Benjamin Chain, John R. Helliwell, Naomi E. Chayen

**Affiliations:** a Division of Systems Medicine, Department of Metabolism, Digestion and Reproduction, Faculty of Medicine, Sir Alexander Fleming Building, Imperial College London, London SW7 2AZ, United Kingdom; b Structural and Supramolecular Chemistry Laboratory, Institute of Nanoscience and Nanotechnology, National Centre for Scientific Research ‘Demokritos’, 15310 Athens, Greece; c Department of Life Sciences, Faculty of Natural Sciences, Sir Ernst Chain Building, Imperial College London, London SW7 2AZ, United Kingdom; d Division of Infection and Immunity, University College London, Gower Street, London, United Kingdom; e Department of Chemistry, The University of Manchester, Manchester M13 9PL, United Kingdom

**Keywords:** CCR5 receptor, RoAb13, HIV entry, PIYDIN, antibodies, structure determination, viruses, X-ray crystallography, structural biology

## Abstract

C-C chemokine receptor 5 (CCR5) is a major co-receptor molecule used by HIV-1 to enter cells. This led to the hypothesis that stimulating an antibody response would block HIV with minimal toxicity. Here, X-ray crystallographic studies of the anti-CCR5 antibody RoAb13 together with two peptides were undertaken: one peptide is a 31-residue peptide containing the PIYDIN sequence and the other is the PIDYIN peptide alone, where PIYDIN is part of the N-terminal region of CCR5 previously shown to be important for HIV entry. In the presence of the longer peptide (the complete N-terminal domain), difference electron density was observed at a site within a hypervariable CDR3 binding region. In the presence of the shorter core peptide PIYDIN, difference electron density is again observed at this CDR3 site, confirming consistent binding for both peptides. This may be useful in the design of a new biomimetic to stimulate an antibody response to CCR5 in order to block HIV infection.

## Abstract 2

C-C chemokine receptor 5 (CCR5) is a major co-receptor molecule used by HIV-1 to enter cells. Two X-ray crystallographic studies are presented of the antibody RoAb13, which binds to the peptide PIYDIN, which is part of the N-terminal domain of CCR5. The results may provide the basis for active immunization vaccines to stimulate an antibody response to native CCR5 that will block HIV infection.

## 1. Introduction   

HIV is a major health problem throughout the world. In 2019, an estimated 38 million people were living with HIV and 690 000 people died of AIDS-related illnesses (with >32.5 million having died since the start of the epidemic). Traditional vaccine approaches continue to be challenging, and the remarkable ability of the virus to mutate may thwart such conventional approaches. In contrast, host-directed therapies may be more robust in the face of viral evolution.

C-C chemokine receptor 5 (CCR5), a G-protein-coupled seven-transmembrane protein, is one of the main entry co-receptors for HIV and blocking it can prevent infection. Individuals who lack CCR5 are resistant to HIV (Gupta *et al.*, 2019 ▸). It is thus an important therapeutic target. The drug maraviroc is used to block the binding of HIV to CCR5. Like all antiretroviral drugs, however, it faces problems of toxicity, drug resistance and the need for long-term administration. CCR5 has also been considered as a potential target for (auto) vaccination, by inhibiting the binding of ligands or by inducing down-regulation of the receptor from the cell surface. However, CCR5 is of course a self-protein, posing significant challenges for vaccine strategies.

An approach to circumventing self-intolerance is to use synthetic peptides mimicking the N-terminal domain of CCR5 together with universal T-cell epitopes to stimulate antibodies against CCR5 (Chain *et al.*, 2008 ▸, 2015 ▸). Although these studies demonstrated that the antipeptide antibody could cross-react with native CCR5 on the cell surface and inhibit viral entry, high concentrations of antibody were required. We hypothesized that this was because the native structure of the CCR5 N-terminal domain was poorly captured by short peptide analogues and that more efficient synthetic immunogens could be designed if the native three-dimensional structure of the N-terminal domain of CCR5 was known. The structure of the N-terminal domain of CCR5 has recently been published by Shaik *et al.* (2019 ▸). We have demonstrated that the monoclonal antibody RoAb13 binds both native CCR5 and a free peptide corresponding to a short sequence of the CCR5 N-terminal domain, and that binding to the receptor blocks HIV replication (Ji *et al.*, 2007 ▸; Chain *et al.*, 2015 ▸). The work presented here reports the conformation of the PIYDIN sequence in the 31-residue peptide analogue of the CCR5 epitope (hereafter referred to more simply as the ‘31 peptide’) bound to RoAb13 by co-crystallizing this synthetic peptide containing the core epitope sequence with the monoclonal antibody RoAb13. The PIYDIN core peptide bound at the same location but with slightly less well formed electron density.

Competitive ELISA testing RoAb13 binding against a synthetic peptide corresponding to the entire N-terminal domain of human CCR5 (hCCR5_1–31_; see Section 2 for the full sequence), as well as against a panel of truncated peptides spanning smaller regions within this domain, showed that the core linear epitope (the minimal sequence required for recognition) spanned the sequence between Pro8 and Asn13, *i.e.* PIYDIN. Further truncation of the first isoleucine and the last asparagine of this sequence completely inhibited binding, whereas truncation of the initial proline inhibited it to an extent (Chain *et al.*, 2015 ▸). It must be noted, however, that PIYDIN (residues 8–13) is only the core epitope for RoAb13 and not generally. For example, for antibody 1801, another anti-CCR5 antibody tested by Chain *et al.* (2015 ▸), the core sequence is the partly overlapping YDINYYT (residues 10–16). For HIV entry, the crucial sequence seems to be YDINYY (residues 10–15, and more specifically YD and YY), but there are some further crucial residues, namely Glu18, Lys21, Gln22 and Gln280 (Farzan *et al.*, 1998 ▸).

The insights gained into the three-dimensional structure of the CCR5 N-terminal domain bound to an anti-CCR5 antibody may inform the design of better peptide analogues for use as immunogens *in vivo*. These analogues may ultimately provide the basis for active immunization vaccines to stimulate an antibody response to native CCR5 that will block HIV infection.

## 2. Methods   

The Fab fragment of RoAb13 and two peptides of lengths 31 residues (the full N-terminal hCCR5_1–31_ loop) and six residues (the core epitope sequence for RoAb13), listed below, were obtained as described by Chain *et al.* (2015 ▸). The peptides were synthesized by Peptide Synthetics (Fareham): MDYQVSSPIYDINYYTSEPCQKINVKQIAAI (31 residues) and PIYDIN. All other reagents and chemicals were of analytical grade and were obtained from Sigma–Aldrich, UK.

### 2.1. Crystallization   

To create complexes of RoAb13 with each peptide, RoAb13 at 5, 8 and 10 mg ml^−1^ in 20 m*M* HEPES pH 7.0, 0.1 *M* NaCl was incubated with 8 m*M* peptide in 0.1 *M* NaOH to give a final peptide concentration of ∼660 µ*M*. Each peptide was incubated with RoAb13 for varying periods between 24 h and seven days before setting up crystallization trials.

RoAb13 complexed with each peptide was screened with Crystal Screen HT and Index (Hampton Research) in sitting-drop vapor-diffusion format using a Mosquito robot (TTP Labtech, UK). All screening trials used 400 nl drops comprised of a 1:1 ratio of protein solution and screen solution. The crystallization trials were incubated at 293 K.

Optimization trials with the 31-peptide complex at 5, 8 and 10 mg ml^−1^ RoAb13 were set up at ammonium sulfate concentrations ranging between 1.5 and 3.0 *M* in increments of 0.5 *M* using both the hanging-drop vapor-diffusion and microbatch methods (Chayen *et al.*, 1992 ▸; Chayen, 1997*b* ▸) at 293 K. The hanging-drop vapor-diffusion trials were carried out using 15-well EasyXtal plates and EasyXtal X-seal screw lids (Qiagen, UK) inverted over 300 µl reservoir solution. Trials with the microbatch method were carried out by mixing equal volumes of the RoAb13–peptide complex and precipitant solutions in 72-well HLA plates from Douglas Instruments (UK) incubated under a layer of paraffin oil (VWR International).

Further fine-tuning of the conditions was performed for the 31-peptide complex with 8 and 10 mg ml^–1^ RoAb13 and 1.5–2.0 *M* ammonium sulfate in increments of 0.1 *M* using the hanging-drop method. 10 µ*M* nickel chloride was used as an additive in further optimization experiments using 1.8–2.0 *M* ammonium sulfate in steps of 0.05 *M*. In addition, a 200 µl oil barrier consisting of a 1:1 ratio of paraffin and silicone oils was introduced over the reservoir solution in the abovementioned conditions (Chayen, 1997*a* ▸). A range of heterogeneous nucleants such as Naomi’s nucleant (Khurshid *et al.*, 2014 ▸), Chayen Reddy MIP (Saridakis & Chayen, 2013 ▸) and PEGylated Carbon Black (Govada *et al.*, 2016 ▸) were also introduced in trials with the 31-peptide complex at 8 and 10 mg ml^−1^ and 1.8 *M* ammonium sulfate. A single grain was introduced in the case of a solid nucleant and a tenth of the total drop volume was used in the case of a liquid nucleant (*i.e.* 0.2 µl of the nucleant in a 2 µl drop).

Trials with the 31-peptide complex were also optimized with 5 mg ml^−1^ RoAb13 and 1.8–2.0 *M* ammonium sulfate in increments of 0.05 *M* by inducing nucleation in a controlled manner and arresting it before excess nucleation occurred (Govada & Chayen, 2009 ▸). 24 h after setting up the experiments, the screw caps of the 15-well EasyXtal plates were loosened for two hours by rotating them by 90° and were then resealed.

In the case of the short peptide (PIYDIN), 10 mg ml^−1^ RoAb13 was incubated for 24 h with peptide stock and the optimization trials for this complex were set up with 1.8–2.0 *M* ammonium sulfate in increments of 0.05 *M* with and without 10 µ*M* nickel chloride.

All optimization trials were performed manually and were observed over a four-week period.

Several soaking experiments were also performed with crystals obtained using 8–10 mg ml^–1^ RoAb13 and 1.8–2.0 *M* ammonium sulfate. These crystals failed to diffract satisfactorily.

### 2.2. Data availability   

RoAb13 co-crystallized with the 31 peptide (with the PIYDIN residues of the 31 peptide included in the model) was deposited in the Protein Data Bank (PDB) as entry 7njz. RoAb13 co-crystallized with PIYDIN was also deposited in the PDB as entry 7nw3. The diffraction images are available at Zenodo as run DLS i04 MX12579-11 Sample 4 (https://doi.org/10.5281/zenodo.4912886) and run DLS i04 MX17221-38 Sample 7 (https://doi.org/0.5281/zenodo.4916326).

## 3. Results   

### 3.1. Crystallization   

Initial crystallization hits were produced with 2.0 *M* ammonium sulfate (Hampton Research Crystal Screen HT condition C8). Both RoAb13–peptide complexes, at an antibody concentration of 10 mg ml^−1^, produced crystals one week after setting up the trials.

**Figure 1:**
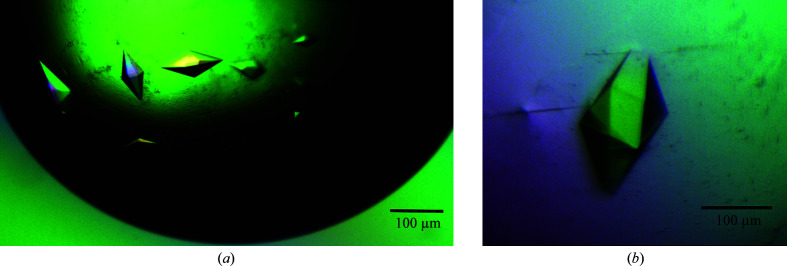
Crystals of RoAb13 complexed with (*a*) the 31 peptide and (*b*) the PIYDIN peptide.

Conventional optimization produced crystals of RoAb13 complexed with the 31 peptide that diffracted to 7 Å resolution at 10 mg ml^−1^ antibody, 2.0 *M* ammonium sulfate, 10 µ*M* nickel chloride [Fig. 1 ▸(*a*)]. Crystals were obtained using both the hanging-drop and microbatch methods.

A portfolio of crystallization methods of optimization, such as slowing down vapor diffusion with an oil barrier (Chayen, 1997*a* ▸) and the use of Naomi’s nucleant (Khurshid *et al.*, 2014 ▸), Chayen Reddy MIP (Saridakis & Chayen, 2013 ▸) and PEGylated Carbon Black (Govada *et al.*, 2016 ▸), showed considerable improvement in crystal diffraction from 7 to 4 Å for RoAb13 complexed with the 31 peptide. The best diffracting crystals (to ∼3.2 Å resolution) of the above complex at an initial concentration of 5 mg ml^−1^ were obtained with 1.85 *M* ammonium sulfate after one week. This was achieved by the induction of nucleation and its subsequent arrest (Govada & Chayen, 2009 ▸).

In the case of RoAb13 complexed with PIYDIN at an initial concentration of 10 mg ml^−1^, the best diffracting crystals (to 3.3 Å resolution) were produced with 1.8 *M* ammonium sulfate after four days in a hanging-drop vapor-diffusion setup [Fig. 1 ▸(*b*)].

### 3.2. X-ray crystallography   

The crystals were cryoprotected with 20% glycerol and data were collected at 100 K on the I04 beamline at the Diamond Light Source (DLS) using a Dectris PILATUS 6M-F detector.

X-ray diffraction data sets were collected from crystals of the antibody co-crystallized with both the 31-residue (long) and the PIYDIN (short) peptides. Two crystals of the 31-peptide complex showed acceptable diffraction and the diffraction images were processed using *iMosflm* (Battye *et al.*, 2011 ▸). The diffraction data were merged with *POINTLESS* and *SCALA* (Evans, 2006 ▸). An attempt was made to combine the data from the two best diffracting crystals, but an *R*_merge_ value in the strongest intensity bin of 11% was obtained, which is of course too large, even though the unit-cell parameters were quite similar (*a* = 76.44, *b* = 76.44, *c* = 268.64 and *a* = 76.53, *b* = 76.53, *c* = 269.07 Å). The *R*_merge_ values in the strongest intensity bin for each crystal individually were 4% and 6%. Keeping the two crystals separate proved an effective approach as comparisons of their omit electron-density maps showed reproducible extended electron density in the same location. (By ‘omit map’, here and subsequently, we mean that the PIYDIN has not been included at this stage of each analysis.) Both crystals show some anisotropic diffraction, which was checked with the UCLA web server (Strong *et al.*, 2006 ▸) and *STARANISO* (Tickle *et al.*, 2018 ▸. Both analyses showed the diffraction resolution to be 3.5 × 3.5 × 2.7 Å with an 〈*I*/σ(*I*)〉 value of 1.5. Amongst many tests, an isotropic resolution cutoff of 3.2 Å proved to be optimal for model refinement. Based on the systematic absences, the space-group symmetry was determined as either *P*4_1_2_1_2 or *P*4_3_2_1_2. In the following, we describe structure solution and refinement using the data set from the slightly better diffracting crystal (Sample 4), which corresponds to the structure deposited in the PDB.

In molecular replacement using *Phaser* (McCoy *et al.*, 2007 ▸), early attempts to place the heavy (H) and light (L) chains of the antibody using the RoAb13 model with PDB code 4s2s (Chain *et al.*, 2015 ▸) did not lead to a satisfactory solution. However, when each of these chains was split into its variable (V) and constant (C) parts (leading to four chains for placement: HV, HC, LV and LC), *Phaser* yielded one clear solution with one copy of each chain in the asymmetric unit. *REFMAC* (Murshudov *et al.*, 2011 ▸) and *phenix.refine* (Afonine *et al.*, 2012 ▸) were used for restrained protein model refinement. Refinement with *phenix.refine* led to an *R* and *R*_free_ of 26.9% and 29.2%, respectively. We have deposited the refined structure in the PDB as entry 7njz (see Table 1 ▸). In the PDB validation report, several C-terminal residues have high RSRZ values, which is due to the intrinsic flexibility of this end of the polypeptide chains: 11.8 for Ile223H, 11.3 for Thr222H and 11.1 for Cys220L.

An X-ray diffraction data set for the PIYDIN co-crystallization was also collected at DLS and evaluated via its pipeline of data-processing software options. The data, collected to 3.2 Å resolution, were processed with *XDS* (Kabsch, 2010 ▸) and scaled with *SCALA* (Evans, 2006 ▸). Again, the crystal belonged to space group *P*4_1_2_1_2, with very similar unit-cell parameters *a* = *b* = 76.6, *c* = 270.1 Å.

Molecular replacement was attempted with both *MOLREP* and *Phaser*, again using PDB entry 4s2s as an initial model. Attempts to separately place the heavy and light chains of the antibody again did not lead to a satisfactory solution, but when each of these chains was split into its variable and constant parts *Phaser* yielded one clear solution with one copy of each chain in the asymmetric unit. Model refinement was undertaken with both *REFMAC* and *phenix.refine* for comparison purposes. Refinement with *phenix.refine* led to an *R* and *R*_free_ of 26.8% and 29.3%, respectively. We have deposited the refined structure in the PDB as entry 7nw3 (see Table 1 ▸).

**Figure 2:**
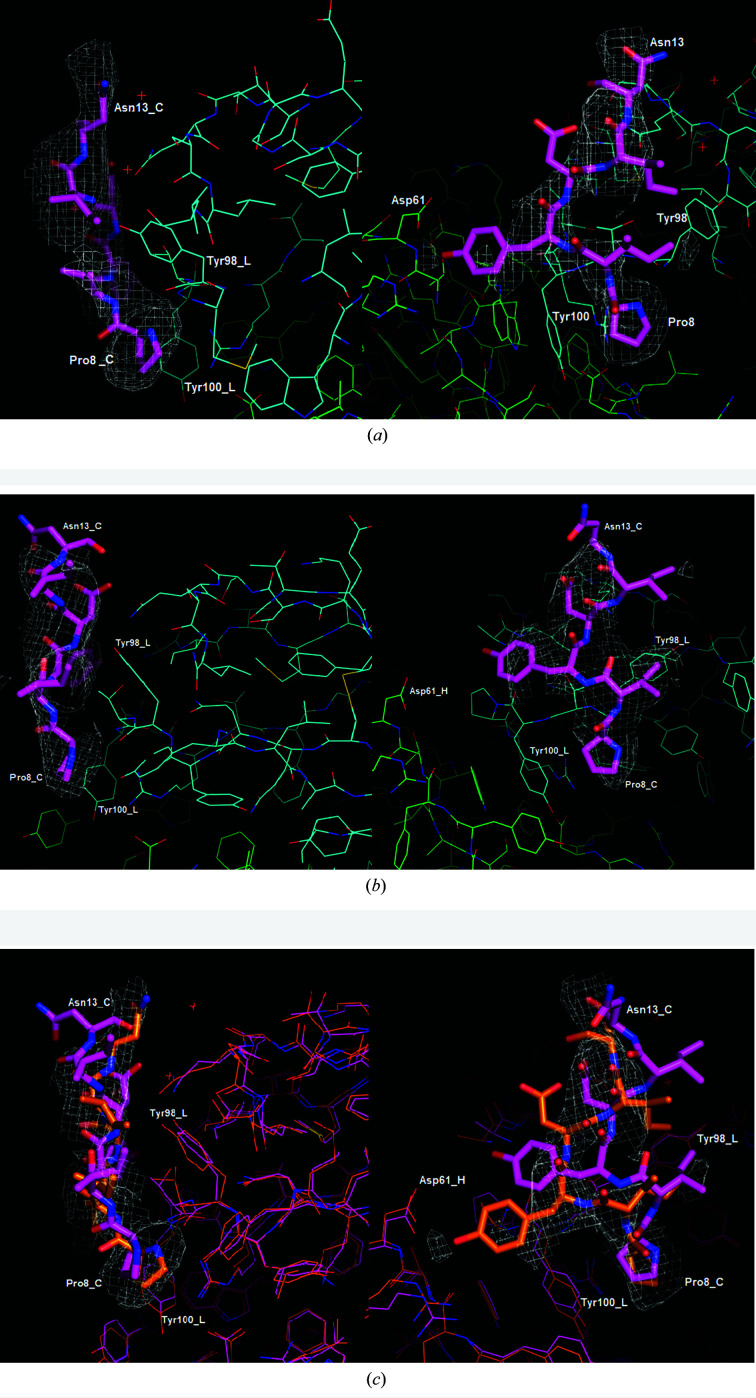
Omit electron-density maps are shown for (*a*) the PIYDIN residues of the 31-peptide study and (*b*) the PIYDIN-only study; orthogonal views are contoured at 2σ in (*a*) and 2.7σ in (*b*). (*c*) shows the overlay of both with the 31-peptide omit map at 2σ; the peptide does not have exactly the same placement but is very similar.

Omit electron-density maps are shown in Fig. 2 ▸ for crystals from both the 31-peptide study and the PIYDIN study. These show the top difference peaks in the peaks list at 6σ, which are in the same location on the antibody surface (see Table 2 ▸). The binding site of PIYDIN also forms a crystal lattice contact with a neighboring protein molecule. The resolution of the data does not permit the accurate reporting of ligand–antibody interactions. However, we observe that in the case of the PIYDIN part of the 31 peptide, Pro8 of the ligand is near (approximately within 3.5 Å of) Tyr98 and Tyr100, Ile9 is near Gly218, and Ile12 is near Tyr98 of the light chain. Tyr10 of the ligand comes near Asp61 and Arg64 of the heavy chain. Asp11 is only engaged in intra-peptide interactions with Ile12 and Asn13. In the PIYDIN-only study, Ile9 additionally comes near Thr99 of the light chain (the corresponding distance is ∼5 Å for the 31-peptide structure). The ligand also approaches symmetry-related residues within the crystal lattice; however, as these belong to the flexible C-terminal tails of the heavy and light chains, which have a poor fit to the map, precision is largely lost (Table 2 ▸).

## 4. Discussion   

From amino-acid sequence comparisons of several hundred different variable domains, the hypervariable antigen-binding regions have been defined as amino acids 24–34 (CDR1), 50–56 (CDR2) and 89–97 (CDR3) in the light chains and 31–35 (CDR1), 50–65 (CDR2) and 95–102 (CDR3) in the heavy chains. These residues occur in the loop regions that connect β-strands. These loop regions are clustered together at one end of the β-sheets. PIYDIN bound to the protein surface near to CDR3 is therefore biologically consistent. The placement of PIYDIN in the two studies is very similar although not identical.

For the remainder of the 31 peptide, at both ends beyond the PIYDIN portion, within this discussion the question arises as to ‘where does the full length go?’. We simply note that PIYDIN binds adjacent to the crystal solvent channels, which could neatly accommodate the remaining portions of the full-length 31 peptide.

## 5. Conclusions   

We have undertaken X-ray crystal structure studies of the antibody RoAb13 with two peptides from the HIV receptor C-C chemokine receptor 5 (CCR5). Co-crystallization of RoAb13 with both peptides was used, rather then the method of soaking a RoAb13 crystal with peptide, which is thus obviously the preferred protocol for the 31 peptide. The key aspects in the 31-peptide study were as follows. Firstly, the *REFMAC* difference omit map showed clear omit electron density. The placement is consistent with truncation experiments showing that removing the PI or the N of PIYDIN abolished binding of the peptide. Indeed, this formed the rationale for choosing PIYDIN rather than any other part of the 31 peptide. Secondly, there is the immunological result that the complementarity-determining CDR3 binding region is known, which is where the omit map density consistent with PIYDIN occurs. Thirdly, for the 31 peptide the answer to ‘where does the full length go?’ is that the PIYDIN binds adjacent to the crystal solvent channels in order for the remaining portions of the full-length 31 peptide to be accommodated. In the sister study, co-crystallization with PIYDIN alone, the largest omit electron density coincides with the PIYDIN placement of the 31 peptide. The pure PIYDIN co-crystallization experiment offers not only reproducibility of the omit map from the 31-peptide experiment and model but also confirmation of the choice of PIYDIN as being the binding portion of the 31 peptide. The binding poses of the two are not identical but are very similar.

These results should be useful in the design of a new biomimetic to stimulate an antibody response to CCR5 in order to block HIV infection.

## Supplementary Material

PDB reference: RoAb13, bound to 31-residue peptide containing the PIYDIN sequence, 7njz

PDB reference: bound to PIYDIN peptide, 7nw3

Run DLS i04 MX12579-11 sample 4: https://doi.org/10.5281/zenodo.4912886

Run DLS i04 MX17221-38 sample 7: https://doi.org/10.5281/zenodo.4916326

## Figures and Tables

**Table 1:** X-ray diffraction data and model-refinement statistics

	Complex with PIYDIN (in the 31 peptide) (PDB entry 7njz)	Complex with PIYDIN alone (PDB entry 7nw3)
Wavelength (Å)	0.97949	0.97949
Crystal-to-detector distance (mm)	590.64	525.90
Crystal angular range (°)	118	180
Angular increment per image (°)	0.1	0.2
No. of images	1180	900
Processing program	*iMosflm*	*XDS*
Data-collection temperature (K)	100	100
Space group	*P*4_1_2_1_2	*P*4_1_2_1_2
Unit-cell parameters (Å)	*a* = *b* = 76.44, *c* = 268.64	*a* = *b* = 76.61, *c* = 270.06
Resolution in last shell (Å)	2.7	3.2
Observed reflections	158879	350702
Unique reflections	22645	14151
Completeness (%)	100.0 (99.9)	99.9 (99.8)
*R*_merge_	0.116 (173.8)	0.179 (6.2)
〈*I*/σ(*I*)〉	7.6 (0.8)	14.3 (0.5)
Multiplicity	7.0 (5.7)	24.8 (26.4)
CC_1/2_	0.998 (0.067)	0.999 (0.529)
Model-refinement resolution (Å)	3.2	3.2
No. of reflections used	13940	14087
Wilson *B* factor (Å^2^)	122.0	98.2
*R* factor/*R*_free_, *REFMAC* (%)	21.6/26.7	
*R* factor/*R*_free_, *phenix.refine* † (%)	26.9/29.2	26.8/29.3
Ramachandran outliers (%)	2.8	0.9
Clashscore	8	14

† *phenix.refine* gave a better rotamer distribution than *REFMAC*, which gave a better *R* and *R*_free_. The PDB deposition was based on the former, albeit with a worse *R*_free_.

**Table 2:** Amino-acid interactions of PIYDIN in the 31-peptide complex (PDB entry 7njz) with RoAb13 These were very similar in the PIYDIN-only study (see footnote).

Amino acid in PIYDIN	Nearest-neighbor amino acids of RoAb13 (within 3.5 Å)
P	**Tyr98L**, **Tyr100L**; Ile223H of symmetry equivalent
I	**Thr99L** †, **Gly218L**; Gly220H, Pro221H of symmetry equivalent
Y	**Asp61H**, **Arg64H**; Cys220L of symmetry equivalent
D	**Ile12C**, **Asn13C**
I	**Tyr98L**
N	**Asp11C**

† This distance was 5 Å in PDB entry 7njz but was 3.5 Å in the PIYDIN-only study (PDB entry 7nw3).

## References

[bb1] Afonine, P. V., Grosse-Kunstleve, R. W., Echols, N., Headd, J. J., Moriarty, N. W., Mustyakimov, M., Terwilliger, T. C., Urzhumtsev, A., Zwart, P. H. & Adams, P. D. (2012). *Acta Cryst.* D**68**, 352–367. 10.1107/S0907444912001308 PMC3322595 22505256

[bb15] Battye, T. G. G., Kontogiannis, L., Johnson, O., Powell, H. R. & Leslie, A. G. W. (2011). *Acta Cryst.* D**67**, 271–281. 10.1107/S0907444910048675 PMC3069742 21460445

[bb2] Chain, B., Arnold, J., Akthar, S., Brandt, M., Davis, D., Noursadeghi, M., Lapp, T., Ji, C., Sankuratri, S., Zhang, Y., Govada, L., Saridakis, E. & Chayen, N. (2015). *PLoS One*, **10**, e0128381. 10.1371/journal.pone.0128381 PMC4451072 26030924

[bb3] Chain, B. M., Noursadeghi, M., Gardener, M., Tsang, J. & Wright, E. (2008). *Vaccine*, **26**, 5752–5759. 10.1016/j.vaccine.2008.08.025 PMC2670972 18765264

[bb4] Chayen, N. E. (1997*a*). *J. Appl. Cryst.* **30**, 198–202.

[bb5] Chayen, N. E. (1997*b*). *Structure*, **5**, 1269–1274. 10.1016/s0969-2126(97)00279-7 9351804

[bb6] Chayen, N. E., Shaw Stewart, P. D. & Blow, D. M. (1992). *J. Cryst. Growth*, **122**, 176–180.

[bb7] Evans, P. (2006). *Acta Cryst.* D**62**, 72–82. 10.1107/S0907444905036693 16369096

[bb8] Farzan, M., Choe, H., Vaca, L., Martin, K., Sun, Y., Desjardins, E., Ruffing, N., Wu, L., Wyatt, R., Gerard, N., Gerard, C. & Sodroski, J. (1998). *J. Virol.* **72**, 1160–1164. 10.1128/jvi.72.2.1160-1164.1998 PMC124591 9445013

[bb9] Govada, L. & Chayen, N. E. (2009). *Cryst. Growth Des.* **9**, 1729–1732.

[bb10] Govada, L., Leese, H. S., Saridakis, E., Kassen, S., Chain, B., Khurshid, S., Menzel, R., Hu, S., Shaffer, M. S. P. & Chayen, N. E. (2016). *Sci. Rep.* **6**, 20053. 10.1038/srep20053 PMC4740738 26843366

[bb11] Gupta, R. K., Abdul-Jawad, S., McCoy, L. E., Mok, H. P., Peppa, D., Salgado, M., Martinez-Picado, J., Nijhuis, M., Wensing, A. M. J., Lee, H., Grant, P., Nastouli, E., Lambert, J., Pace, M., Salasc, F., Monit, C., Innes, A. J., Muir, L., Waters, L., Frater, J., Lever, A. M. L., Edwards, S. G., Gabriel, I. H. & Olavarria, E. (2019). *Nature*, **568**, 244–248. 10.1038/s41586-019-1027-4 PMC7275870 30836379

[bb12] Ji, C., Brandt, M., Dioszegi, M., Jekle, A., Schwoerer, S., Challand, S., Zhang, J., Chen, Y., Zautke, L., Achhammer, G., Baehner, M., Kroetz, S., Heilek-Snyder, G., Schumacher, R., Cammack, N. & Sankuratri, S. (2007). *Antiviral Res.* **74**, 125–137. 10.1016/j.antiviral.2006.11.003 17166600

[bb13] Kabsch, W. (2010). *Acta Cryst.* D**66**, 125–132. 10.1107/S0907444909047337 PMC2815665 20124692

[bb14] Khurshid, S., Saridakis, E., Govada, L. & Chayen, N. E. (2014). *Nat. Protoc.* **9**, 1621–1633. 10.1038/nprot.2014.109 24922271

[bb16] McCoy, A. J., Grosse-Kunstleve, R. W., Adams, P. D., Winn, M. D., Storoni, L. C. & Read, R. J. (2007). *J. Appl. Cryst.* **40**, 658–674. 10.1107/S0021889807021206 PMC2483472 19461840

[bb17] Murshudov, G. N., Skubák, P., Lebedev, A. A., Pannu, N. S., Steiner, R. A., Nicholls, R. A., Winn, M. D., Long, F. & Vagin, A. A. (2011). *Acta Cryst.* D**67**, 355–367. 10.1107/S0907444911001314 PMC3069751 21460454

[bb18] Saridakis, E. & Chayen, N. E. (2013). *Trends Biotechnol.* **31**, 515–520. 10.1016/j.tibtech.2013.05.003 23764007

[bb19] Shaik, M. M., Peng, H., Lu, J., Rits-Volloch, S., Xu, C., Liao, M. & Chen, B. (2019). *Nature*, **565**, 318–323. 10.1038/s41586-018-0804-9 PMC6391877 30542158

[bb20] Strong, M., Sawaya, M. R., Wang, S. S., Phillips, M., Cascio, D. & Eisenberg, D. (2006). *Proc. Natl Acad. Sci. USA*, **103**, 8060–8065. 10.1073/pnas.0602606103 PMC1472429 16690741

[bb21] Tickle, I. J., Flensburg, C., Keller, P., Paciorek, W., Sharff, A., Vonrhein, C. & Bricogne, G. (2018). *STARANISO*. Global Phasing Ltd, Cambridge, United Kingdom.

